# Commentary: Genomic, epigenomic, and transcriptomic signatures of prostate cancer between African American and European American patients

**DOI:** 10.3389/fonc.2023.1218669

**Published:** 2023-08-22

**Authors:** Francois G. Rollin, Sudarshan Krishnamurthy, Surabhi Beriwal

**Affiliations:** ^1^ Department of Medicine, Emory University School of Medicine, Atlanta, GA, United States; ^2^ Wake Forest School of Medicine, Winston-Salem, NC, United States; ^3^ Emory University School of Medicine, Atlanta, GA, United States

**Keywords:** prostate cancer, health disparities, cancer genetics, epidemiology, equity

The recent review article by Stevens and colleagues (February 28) (1) explores the genomic, epigenomic, and transcriptomic signatures of prostate cancer in racialized groups. We agree that racial inequities in prostate cancer incidence and outcome need to be described and urgently addressed in order to achieve health equity. However, we have several concerns regarding the methods used and the possible implications of inherent genetic racial differences. We recognize that the appropriate use of race in research is complicated and that we are all learning to do this better with the shared goal of reducing health disparities.

Since racial categories are socio-political constructs and poor proxies for human genetic variation (2, 3), the rationale for the use of these categories must be clearly explained in the methods to avoid inadvertently perpetuating the myth of race as an inherent biological category.

The *apriori* stratification of cases by racial category in the analysis in genomic studies, while unfortunately still common, can introduce bias (4). This can be difficult when reviewing at previously published studies, but we should be clear going forward that there is no scientific reason to keep genomic data segregated by racial category (4) unless it is to look at how racism might lead to differences (4). A recent review points out that “[m]ost somatic genome defects are shared between prostate cancers from Black and White men” (5) and describes the fact that “chronic or recurrent prostate inflammation is likely an important driver of neoplastic transformation and malignant progression in the gland.” (5)

In addition, we believe that the use of the categories “African American” and “European American” mixes both self-identified race and the genetic ancestry construct of ‘European American.’ This introduces ambiguity and confusion in the population descriptors and could inappropriately imply categorical differences, when genetic differences are in fact gradual or clinal (4). In the discussion, the authors claim that it is “important to confirm and measure ethnicity information from samples using genetic ancestry informative marker data.” (1) The delineation between ancestry, genetic ancestry, and genetic similarity is complicated but important to state clearly (4, 6). Neither “race” nor “ethnicity” can be confirmed or measured by so called ‘ancestry informative markers,’ (6) and the use of continental ancestry designations is not consistent with modern understanding of genetic variation in humans.

The National Academy of Science recently published a report outlining a new framework for the use of population descriptors in genomic research (4). In this report, the first recommendation is that “researchers should not use race as a proxy for human genetic variation. In particular, researchers should not assign genetic ancestry group labels to individuals or sets of individuals based on their race, whether self-identified or not.” (4)

The authors begin the review article by stating that the differences in the prostate cancer incidence and outcome between these groups “appear to be attributable to socioeconomic factors” (1) but they continue “in addition to socioeconomic factors, biological factors may further widen the gap.” (1) The authors appropriately state that “additional large-scale investigations that take into account of potential confounding factors are greatly needed,” (1) but the paper does not explicitly consider sources of residual confounding in their discussion of genomic associations between racial categories and outcomes.

One 2021 study found that “contemporary next-generation sequencing of primary and metastatic prostate cancer did not reveal any significant differences in actionable mutations between self-reported races”. (5) If genes (FOXA1, KMT2D, SPOP, MYC, PTEN, TP53, ZFHX3, and TMPRSS2-ERG) (1) are associated with an outcome we can learn about biologic pathways of disease. But we cannot claim that a social constructed racial category that may be correlated with some of these genes is the cause of that difference. This distinction is especially important to prevent the misattribution of differences to race rather than racism (7), distracting from the root cause of inequities.

The observed differences between racial categories are almost completely due to residual confounding and embodiment of inequality (or allostatic load (8)). Therefore, the effects of structural racism must be assessed or if that is not possible it must be mentioned as a limitation. For example, there is evidence of dietary factors that explain some of the observed racial and socioeconomic differences in prostate cancer. In addition, we note that not using the word racism in this review paper when there is clear evidence of the role of structural racism in prostate cancer mortality disparity (3, 9, 10) could lead to misattribution of causation—it could imply that there exist biological differences among racial categories that are strictly social constructs.

While we are not questioning the intentions of the authors, we are strongly advocating for the naming of systemic and structural racism, and not race itself, as the root cause of racial inequity in prostate cancer (10, 11).

## Author contributions

FR wrote the first draft of the manuscript. FR, SK, and SB all revised the manuscript and approved the submitted version.
